# Changes and Risk Factors of Skeletal Muscle Mass and Strength in Patients with Type 2 Diabetes over 60 Years Old: A Cross-Sectional Study from China

**DOI:** 10.1155/2020/9815485

**Published:** 2020-09-25

**Authors:** Liming Hou, Yan Liu, Xing Li, Cong Huo, Xin Jia, Jie Yang, Yunzhen Lei, Rong Xu, Chao Sun, Xiaoming Wang

**Affiliations:** ^1^Department of Geriatrics, Xijing Hospital, Fourth Military Medical University, Xi'an, Shaanxi Province 710032, China; ^2^Clinical Research Center for Geriatric Diseases of Shaanxi Province, Xi'an, Shaanxi Province 710032, China

## Abstract

**Objective:**

The accelerate loss of skeletal muscle mass, strength, and function, named sarcopenia, is a progressive and generalised skeletal muscle disorder, and it is always associated with increased outcomes including falls, frailty, and disability. Diabetes mellitus is associated with significant muscle and physical complications. We aimed at clarifying the changes and risk factors of skeletal muscle mass and strength in elderly with type 2 diabetes.

**Methods:**

The study consisted of patients with type 2 diabetes (*n* = 120) and an older general population (*n* = 126). The skeletal muscle mass and muscle strength, as well as the serum levels of chronic inflammation, oxidative stress, homocysteine, and insulin-like factor-1 were assessed, and the correlation and regression analysis were conducted to evaluate outcomes.

**Results:**

T2DM patients exhibited lower muscle strength compared with the non-T2DM subjects (*P* < 0.01). Among T2DM patients, serum IGF-1 levels were positively correlated with muscle strength (*r* = 0.255, *P* < 0.01) and muscle mass (*r* = 0.209, *P* < 0.05), levels of 8-OHdG were inversely correlated with muscle strength (*r* = −0.252, *P* < 0.01), and there was a negative association between HCY and muscle mass (*r* = −0.185, *P* < 0.05). Muscle mass and strength of patients with higher education level were significantly higher than those with lower education level (*P* < 0.05), in male patients, muscle mass and muscle strength were significantly lower in smokers (*P* < 0.01), and muscle mass was lower in chronic drinkers (*P* < 0.05).

**Conclusions:**

These findings suggest that diabetic patients may be more susceptible to sarcopenia at an older age. And it also provides evidences that among elderly with diabetes mellitus, oxidative damage and HCY as well as IGF-1 are important predictors of age-dependent sarcopenia.

## 1. Background

The explosion of aging population represents a worldwide demographic phenomenon which has put modern society under great challenge. Aging is accompanied by the high prevalence of age-related chronic diseases and geriatric conditions. In this sense, age-related changes in body composition are especially important that muscle is essential for locomotion, and it can negatively affect functional status in older adults, including a progressive decrease in muscle mass and strength, as well as function. The term of sarcopenia refers to the muscle loss that occurs with aging [1]. Explorative study has shown that sarcopenia and frailty share the same biomedical determinants (aging, disease, inflammation, hormonal deficiencies, and malnutrition). The presence of sarcopenia correlates with functional impairment and adverse health outcomes, providing the background for subsequent disability, hospitalization, loss of independence, and frailty [2]. Studies suggest that sarcopenia could be an important early characteristic for the onset of frailty; sarcopenia and frailty are associated with one another and often coexist [3]. Edmonton FRAIL scale had been proved useful in determining the frail subjects [4, 5]. However, the complexity and the multidimensional character of sarcopenia make the prognosis difficult. Furthermore, diabetes has been associated with a two to threefold increased risk of developing physical disability in elderly [6]. Moreover, sarcopenia was recognized as the third most prevalent complication of diabetes. It is recognized that the presence of sarcopenia has an adverse impact on diabetes progress; it conferred a greater risk of fall, fracture, and disability in T2DM. Therefore, it is clinically of great importance to have detailed knowledge about the occurrence of sarcopenia in elderly T2DM patients [7].

The etiology of muscle weakness in T2DM patients remains unclear; several factors such as chronic inflammation, nutritional changes, life-style modification, oxidative stress, and hormonal changes maybe intervene in. Cross-sectional studies have reported that in the general populations, low-grade chronic inflammation has a connection with the age-related sarcopenia [8]. On the other hand, oxidative stress serves as a key regulator of cell signaling pathways, which may contribute to low muscle mass; thus, changes in oxidative stress play important roles in the development of sarcopenia. 8-hydroxy-2′-deoxyguanosine (8-OHdG), the most frequently used biomarker of end product of oxidative DNA damage, is continuously produced in living cells, and emerging evidence has implicated that the elevated 8-OhdG may be related to cancers, cardiovascular diseases, COPD, and several degenerative diseases. However, the level of 8-OhdG in sarcopenia is still unclear [9]. Furthermore, few studies have attempted to assess the relationships between 8-OHdG level and poor skeletal muscle mass and strength. HCY is the sulfur-containing ammonia produced by methionine in the metabolic process; there are strong evidences that elevated HCY has an adverse effect on multiple organs including vascular, neurologic, and skeletal system. Experimental evidence has revealed that hyper homocysteine (HHCY) plays a causal role in the decreased physical function through mitochondrial dysfunction, as well as epigenetic changes [10]. Analysis from the B-PROOF study suggested that HHCY is a risk factor of the reduced muscle strength and physical performance in the old women; it is a known risk factor for the fractures, disability, frailty, and poor physical function; thus, the clinical significance of HCY in sarcopenia warrants further investigation [11]. In addition, insulin-like growth factor-1 (IGF-1) is a single-chain polypeptide that was mainly synthesize in liver and kidney, which has the functions of anti-inflammatory, lowering blood glucose, promoting growth, cell differentiation, and proliferation, as well as inhibiting apoptosis; it is also a potent anabolic hormone mediating muscle growth, hypertrophy, and regeneration [12]. Recent evidences have suggested that the age-dependent decrease of the IGF-1 level is a major endocrine defect implicated in frailty, disability, and mortality in the elderly [13]. However, the role of IGF-1 in age-related muscle wasting is still unclear and is the focus of present discussion [14].

Therefore, a cross-sectional, controlled observational study was performed to evaluate the muscle mass and strength in older adults with and without diabetes and to have a better understanding of the underlying mechanism among them.

## 2. Methods

### 2.1. Study Population

We recruited 120 participants with T2DM aged 60 years or older, who admitted to the department of endocrinology of Xijing Hospital (Xi'an, China) between January 2019 and April 2019. The participants were excluded if they were accompanied by one or more of the following conditions: (1) have serious bone and joint disease or neuromuscular disease that affect the daily activities; (2) patients with a history of serious physical impairment, such as acute cerebrovascular events, gastrointestinal bleeding, sepsis, acute kidney failure, acute coronary syndrome, acute liver failure, and acute respiratory failure; (3) serious diabetic complications, such as severe diabetic nephropathy and diabetic retinopathy and diabetic foot gangrene; (4) accompanied with similar diseases such as malignant tumors. In addition, we enrolled 126 elderly non-T2DM volunteers at the health examination center in the same period.

We gathered the basic information including sex, age, smoking, alcohol consumption, education, dietary habit, sleep patterns, the form of physical activity, and duration time. This study was approved by the Ethics Committee of the First Affiliated Hospital of the Fourth Military Medical University (No.KY20192010-F-1), and it conforms to the provisions of the Declaration of Helsinki, and all the subjects have been given informed consent, and patient anonymity has been preserved.

### 2.2. Measurements

#### 2.2.1. Muscle Mass

A body composition analyzer (TanitaMC) was used to evaluate the appendicular skeletal muscle mass (ASMM), as well as the body weight. Details of the subjects were entered into the machine, including age and sex, then the subjects were asked to stand on the footpads with bare feet and grasped the handles, under a relax and comfortable circumstance. We also calculated the body mass index (BMI) as the supplementary indicators of body composition. For BMI, it is the ratio of body weight to the square of height (kg/m^2^).

#### 2.2.2. Muscle Strength

We performed grip strength measurement as an indicator of the overall muscle strength. A Jamar Electric Grip Dynamometer was used to measure the handgrip strength. The participants were asked to sit down and with their elbow flexed at an angle of 90°. The grip strength measurement was performed three times for each hand and defined as the best score of the left and right hand.

#### 2.2.3. Sample Collection and Biochemical Measurement

The fasting HbA_1_C, VitD_3_, and calcium levels were measured, respectively, in the automatic biochemical analyzer. The next day after admission, 3 ml of the patient's fasting venous blood was extracted, and serum specimens were separated (set the temperature of 4°C, 3000 x g centrifuge for 10 min), then save to -80°C refrigerator under test. Serum IL-6, TNF-*α*, 8-OHdG, HCY, and IGF-1 levels were detected through ELISA kits (Cloud-Clone Corp).

### 2.3. Statistical Analysis

All the continuous variables were presented as the means and standard deviations (^−^x ± s); log transformation will be performed if the data are not normally distributed; two sample *t*-tests were used for intergroup comparison. Categorical variables were expressed by percentage (%) and chi-squared test was used for comparison between groups. Pearson and Spearman correlation analysis was used for correlation tests. Multivariate stepwise regression was used in the multivariate regression analysis. All the calculations are performed with the SPSS 20.0 software; *P* < 0.05 was considered statistically significant.

## 3. Results

### 3.1. The Basic Information of the Subjects

The baseline characteristics of the participants including 120 diabetics and 126 NDM subjects are shown in Table 1. The mean age of the participants in the two groups was 66.89 ± 5.49 y and 66.87 ± 5.00 y, respectively, and there were no significant differences in the age, gender, smoking, drinking, and diet (*P* > 0.05). HbA_1_C of T2DM patients was 8.88 ± 2.41%, significantly lower than that of the NDM (5.66 ± 0.38%) (*P* < 0.01). Calcium and VitD_3_ were 2.23 ± 0.20 mmol/L and 39.98 ± 20.73 ng/ml in the T2DM patients, significantly lower than that of the NDM (2.35 ± 0.14 mmol/L, 49.05 ± 18.16 ng/ml) (*P* < 0.01).

### 3.2. Comparison of Skeletal Muscle Mass, Strength, and Biomarkers between T2DM Patients and NDM Controls

The muscle strength was lower among T2DM patients than NDM controls (25.03 ± 7.85 vs. 29.52 ± 7.73 kg, *P* < 0.01), although no significant difference was noted in ASMM between T2DM and NDM groups (Table 1).

The serum level of 8-OHdG and HCY was higher among T2DM subjects compared to NDM subjects (8-OHdG: 3.08 ± 0.26 vs. 2.59 ± 0.16 pg/ml, *P* < 0.01; HCY: 1.21 ± 0.43 vs. 0.91 ± 0.63 nmol/ml, *P* < 0.01). IGF-1 levels were higher in NDM than that of T2DM subjects (164.39 ± 84.59 vs. 137.11 ± 73.32 ng/ml, *P* < 0.05). However, no difference was observed for the levels of IL-6 and TNF-*α* between the two groups (Table 1).

### 3.3. Factors Associated with Skeletal Muscle Mass and Muscle Strength

We attempted to determine the relationship between the skeletal muscle mass and muscle strength among T2DM patients; height, weight, 8-OHdG, and IGF-1 levels were found to display a significant relationship with grip strength in T2DM patients. In addition, height, weight, BMI, HCY, and IGF-1 were shown in connection with skeletal muscle mass (Table 2).

### 3.4. Effects of Lifestyle on Skeletal Muscle Mass and Muscle Strength in T2DM Patients

To examine the effects of diet, education level, daily activities, and lifestyle on skeletal muscle mass and muscle strength of elderly T2DM patients, participants with diabetes were divided into two groups according to diet, education level, activity, smoking, and alcohol drinking. We failed to note any difference in skeletal muscle mass and muscle strength among T2DM participants with different dietary, sleep, and activity conditions (*P* > 0.05). Interestingly, skeletal muscle mass and muscle strength were found to be much higher in those with higher education level compared with lower education level (*P* < 0.05). Smoking and drinking status of male patients were grouped, and their skeletal muscle mass and muscle strength were compared; the results showed that the skeletal muscle mass and muscle strength of smokers in male patients were significantly lower than that of nonsmokers (*P* < 0.01), and the skeletal muscle mass of drinkers was significantly lower than that of nondrinking (*P* < 0.05) (Table 3).

### 3.5. Multivariate Regression Analysis of the Risk Factors of the Skeletal Muscle Mass and Strength in T2DM Patients

To determine which factors may be independently related to skeletal muscle mass and strength, we conducted multivariate regression models testing the association of several factors with skeletal muscle mass and muscle strength. We employed muscle strength as the dependent variable, gender, height, weight, education level, 8-OHdG, and IGF-1 as the independent variables. In the T2DM patients, the 8-OHdG level was negatively related to muscle strength, and height was related to muscle strength in a positive manner (*r*^2^ = 0.457).

We also conducted the regression analysis for skeletal muscle mass, in the model using height, weight, BMI, HCY, and IGF-1 as the independent variables; we find that the weight and height are the risk factors of the low skeletal muscle mass (*r*^2^ = 0.822).

## 4. Discussion

In the current study, the average level of skeletal muscle mass and strength as well as the risk factors were evaluated in elderly T2DM patients. Our data noted that older adults with T2DM displayed a lower muscle strength and higher 8-OHdG and HCY, as well as a decreased serum IGF-1 levels compared with those of nondiabetics. Furthermore, changes of these biomarkers were correlated with muscle mass and strength in the elderly T2DM patients. Considering the importance of diabetes and sarcopenia, we speculate that early management of skeletal muscle in the elderly T2DM patients may shed some promises towards reducing adverse events, thus promote the life quality and survival of the elderly.

Targeting sarcopenia in diabetics has become an attractive approach given the high incidence of sarcopenia in diabetes. In a cross-sectional study involving 69 T2DM patients, Ogama and colleagues reported a 2.7% incidence of sarcopenia in the elderly T2DM patients with normal cognition and a significant rise of 11.6% in patients with cognitive impairment [15]. Independent study from Leenders and coworkers suggested that diabetes was associated with an approximately 3% lower ASMM in the elderly [16]. Sarcopenia and frailty are associated with one another and often coexist; studies have shown that the prevalence of frailty among T2DM patients was 28.8%, and there was 42.5% overlap between frailty and sarcopenia [17]. In our study, we did not find a difference of muscle mass between diabetics and nondiabetics; this might be due to the patients we enrolled are younger, better glycemic controlled compared with the previous studies.

Janssen et al. reported that weight and height could explain 50% of the variance in skeletal muscle mass in both genders; this might be explained that taller subjects have longer bones and muscles, and heavier subjects require more muscles for their daily activities [18]. Past studies have linked lower education level with the poor control of the glucose and higher mortality of diabetes [19]. In our analysis, we found that education level was negatively associated with the skeletal muscle mass and muscle strength of the diabetics for the first time. We also found that there exists a negative correlation between smoking with skeletal muscle mass and muscle strength in the male; this is in consistent with the Rancho cohort study based on 845 male [20], which reported that smoking is a strong risk factor of sarcopenia [21], and smoking accelerates the loss of skeletal muscle which stimulates protein breakdown and inhibits protein synthesis [22]. In addition, studies have demonstrated that diabetes patients who have a history of drinking had a lower muscle strength; this contrasts with the prior study that showed handgrip strength was positively associated with alcohol consumption, which declared that the individuals who consume moderate amounts of alcohol may be physically active and have greater muscle strength [23].

A previous review showed that oxidative stress plays an important role in the aging process of skeletal muscle [24]. Mecocci et al. showed the age-dependent increase in oxidative damage to DNA, lipids, and protein in human skeletal muscle; the findings suggest that oxidative damage plays an important role in the decline of maximal functional activity during the aging process of human skeletal muscle [25]. Data obtained from male skeletal muscle indicate an increase in DNA damage during aging [26]; however, few studies have investigated the relationship between DNA damage and skeletal muscle in T2DM patients; in our study, this has been demonstrated for the first time.

Another noteworthy finding of this study is the inverse relationship between HCY and muscle mass. Past studies have linked HCY with several diseases, including cardiovascular disease, atherosclerosis, and thrombosis [27]. Kuo et al. showed that elevated HCY is associated with disability in the elderly; the HCY level may be an important indicator of performance status in older adults [28]. Cross-sectional and 3-year follow-up data from the LASA (Longitudinal Aging Study Amsterdam) reported that there exists an inverse association of HCY level with functional limitations in older men and women and a negative relationship with muscle strength in older men [29]. Ng et al. similarly supported that HCY level is correlated with the physical and functional decline of the elderly [30].

Previous studies showed that there was a negative relationship between IGF-1 level and age; cross-sectional analysis from InCHIANTI cohort (Invecchiare in Chianti) showed that IGF-1 is an important modulator of muscle mass and function across the entire life span [31]. Mecocci et al., using the WHAS (Women's Health and Aging Study), identified that a lower level of IGF-1 predicted a greater self-reported difficulty in mobility tasks, poorer knee extensor strength, and slower walking speed in elderly women [25]. In elderly men, Papadakis et al. similarly found that IGF-1 level is positively correlated with the muscle strength and function; however, after adjusting for age, there was no association of IGF-1 levels to functional status independent of age [32]. Kaplan et al. conducted a cohort study with 1122 individuals aged 65 or older, indicated that lower IGF-1 had a significant association with worse handgrip strength, and high IGFBP-1 levels were associated with greater risk of mortality and poor function ability [33]. In the present study, we found a positive association between IGF-1 with muscle mass and muscle strength, but the association is poor after adjustment for gender, height, and so on.

Nevertheless, this study suffers from several limitations. First, this is a cross-sectional study from single department, and although our sample size was comparable to previous studies, additional large multicenter cohort studies with appropriate sample size and longitudinal investigation are warranted in the future. Second, grip strength was used in this study to evaluate muscle strength; although it has been proved to be more accurately represents muscle strength in the human body, but knee extension strength and leg press strength are also available; therefore, a more in-depth comparison between several muscle strengths is needed. Third, due to the limitation of experimental conditions, muscle function detections were not carried out in this study; thus, muscle function should be further explored in future studies.

In conclusion, the findings of this study suggest that diabetic elderly may be more susceptible to sarcopenia, and skeletal muscle mass and strength measuring may be a useful tool in the management of T2DM patients. Furthermore, these findings suggest that changes in oxidative stress, HCY, and IGF-1 and the anthropometric indicators including height, weight, and BMI, as well as the life habit (education, smoking, and drinking) could be the risk factors and diagnostic marker of sarcopenia in the TDM elderly.

## Figures and Tables

**Table 1:** The basic information of the subjects (mean ± SD or %).

	T2DM (*n* = 120)	NDM (*n* = 126)	*P*
Basic information			
Gender (male/female)	69/51	70/56	0.759
Age (y)	66.86 ± 5.49	66.87 ± 5.00	0.992
Smoking			
[*n* (%)]	37 (30.8)	34 (27.0)	0.574
Male/female	37/0	34/0	
Drinking			
[*n* (%)]	31 (25.8)	29 (23.0)	0.657
Male/female	31/0	29/0	
Balanced diet [*n* (%)]	69 (57.50)	58 (46.03)	0.072
Sleeping >5 h [*n* (%)]	82 (68.33)	73 (57.94)	0.091
Physical activity [>1 h/d, *n* (%)]	71 (59.17)	63 (50.0)	0.149
Education level [>8 y, *n* (%)]	75 (62.5)	69 (54.76)	0.218
Anthropometric measurements			
Height (cm)	163.78 ± 8.17	165.70 ± 7.47	0.057
Weight (kg)	65.77 ± 11.61	65.45 ± 9.77	0.819
BMI (kg/m^2^)	24.45 ± 3.47	23.78 ± 2.64	0.088
Grip strength (kg)	25.03 ± 7.85	29.52 ± 7.73	<0.01^∗∗^
ASMM (kg)	21.36 ± 5.46	22.01 ± 5.22	0.998
Blood tests			
HbA_1_C (%)	8.88 ± 2.41	5.66 ± 0.38	<0.01^∗∗^
Calcium (mmol/L)	2.23 ± 0.20	2.35 ± 0.14	<0.01^∗∗^
VitD_3_ (ng/ml)	39.98 ± 20.73	49.05 ± 18.16	<0.01^∗∗^
lg(IL-6) (pg/ml)	1.20 ± 0.27	1.23 ± 0.41	0.366
lg(TNF-*α*) (pg/ml)	1.43 ± 0.55	1.29 ± 0.57	0.087
lg(8-OHdG) (pg/ml)	3.08 ± 0.26	2.59 ± 0.16	<0.01^∗∗^
lg(HCY) (nmol/ml)	1.21 ± 0.43	0.91 ± 0.63	<0.01^∗∗^
IGF-1 (ng/ml)	137.11 ± 73.32	164.39 ± 84.59	<0.05^∗^

**Table 2:** Correlation analysis between skeletal muscle mass and strength with several factors.

	Grip strength (*n* = 120)		ASMM (*n* = 120)	
	*r*	*P*	*r*	*P*
Age (y)	-0.136	0.137	-0.088	0.339
Height (cm)	0.644^∗∗^	0.000	0.841^∗∗^	0.000
Weight (kg)	0.456^∗∗^	0.000	0.768^∗∗^	0.000
BMI (kg/m^2^)	0.109	0.234	0.362^∗∗^	0.000
HbA_1_C (%)	-0.011	0.902	0.099	0.289
VitD_3_ (ng/ml)	0.142	0.122	0.178	0.052
Calcium (mmol/L)	0.136	0.139	0.128	0.164
lg(IL-6) (pg/ml)	-0.109	0.237	-0.072	0.433
lg(TNF-*α*) (pg/ml)	-0.051	0.583	-0.021	0.824
lg(8-OHdG) (pg/ml)	-0.252^∗∗^	0.006	-0.173	0.059
lg(HCY) (nmol/ml)	-0.161	0.080	-0.185^∗^	0.044
IGF-1 (ng/ml)	0.255^∗∗^	0.005	0.209^∗^	0.022

**Table 3:** The skeletal muscle mass and strength in T2DM patients with different lifestyle.

Outcome	Subgroup		*n*	Mean ± SD	*P*
Grip strength (kg)	Diet	Balanced	69	25.36 ± 7.80	0.600
		Unbalanced	51	24.59 ± 7.98	
	Sleep	>5 h/d	82	25.46 ± 7.94	0.728
		<5 h/d	32	24.88 ± 7.92	
	Activities	Regularly, >1 h/d	71	25.46 ± 7.28	0.467
		Irregular	49	24.40 ± 8.64	
	Education level	>8 y	75	26.25 ± 6.96	<0.05^∗^
		8 y or less	45	23.00 ± 8.86	
	Smoking (male)	Smoking	37	27.55 ± 5.33	<0.01^∗∗^
		Nonsmoking	32	31.96 ± 5.87	
	Drinking (male)	Drinking	31	28.65 ± 6.43	0.235
		Nondrinking	38	30.37 ± 5.55	

ASMM (kg)	Diet	Balanced	69	22.02 ± 5.29	0.122
		Unbalanced	51	20.46 ± 5.62	
	Sleep	>5 h/d	82	21.53 ± 5.38	0.951
		<5 h/d	32	21.45 ± 6.02	
	Activities	Regularly, >1 h/d	71	21.58 ± 5.26	0.585
		Irregular	49	21.03 ± 5.78	
	Education level	>8 y or more	75	22.21 ± 5.02	<0.05^∗^
		<8 y	45	19.93 ± 5.91	
	Smoking (male)	Smoking	37	24.34 ± 2.07	<0.01^∗∗^
		Nonsmoking	32	26.84 ± 2.39	
	Drinking (male)	Drinking	31	24.68 ± 2.65	<0.05^∗^
		Nondrinking	38	26.16 ± 2.27	

## Data Availability

The data used to support the findings of this study are available from the corresponding author upon request.
